# Development and piloting of a perturbation stationary bicycle robotic system that provides unexpected lateral perturbations during bicycling (the PerStBiRo system)

**DOI:** 10.1186/s12877-021-02015-1

**Published:** 2021-01-21

**Authors:** Shani Batcir, Yaakov Livne, Rotem Lev Lehman, Shmil Edelman, Lavi Schiller, Omri Lubovsky, Guy Shani, Amir Shapiro, Itshak Melzer

**Affiliations:** 1 Department of Physical Therapy, Recanati School for Community Health Professions, Faculty of Health Sciences, Ben-Gurion University of the Negev, P.O.B. 653, 84105 Beer-Sheva, Israel; 2 Department of Mechanical Engineering, Faculty of Engineering, Ben-Gurion University of the Negev, P.O.B. 653, 84105 Beer-Sheva, Israel; 3 Department of Software and Information Systems Engineering, Faculty of Engineering Sciences, Ben-Gurion University, Beer-Sheva, Israel; 4 Department of Orthopedic Surgery, Barzilai Medical Center, Ashkelon, Israel

**Keywords:** Balance control, Balance reactive responses, Falls, Fall-prevention training, Perturbation training, Old people

## Abstract

**Background:**

Balance control, and specifically balance reactive responses that contribute to maintaining balance when balance is lost unexpectedly, is impaired in older people. This leads to an increased fall risk and injurious falls. Improving balance reactive responses is one of the goals in fall-prevention training programs. Perturbation training during standing or treadmill walking that specifically challenges the balance reactive responses has shown very promising results; however, only older people who are able to perform treadmill walking can participate in these training regimes. Thus, we aimed to develop, build, and pilot a mechatronic Perturbation Stationary Bicycle Robotic system (i.e., PerStBiRo) that can challenge balance while sitting on a stationary bicycle, with the aim of improving balance proactive and reactive control.

**Methods:**

This paper describes the development, and building of the PerStBiRo using stationary bicycles. In addition, we conducted a pilot randomized control trial (RCT) with 13 older people who were allocated to PerStBiRo training (*N* = 7) versus a control group, riding stationary bicycles (*N* = 6). The Postural Sway Test, Berg Balance Test (BBS), and 6-min Walk Test were measured before and after 3 months i.e., 20 training sessions.

**Results:**

The PerStBiRo System provides programmed controlled unannounced lateral balance perturbations during stationary bicycling. Its software is able to identify a trainee’s proactive and reactive balance responses using the Microsoft Kinect™ system. After a perturbation, when identifying a trainee’s trunk and arm reactive balance response, the software controls the motor of the PerStBiRo system to stop the perturbation. The pilot RCT shows that, older people who participated in the PerStBiRo training significantly improved the BBS (54 to 56, *p* = 0.026) and Postural Sway velocity (20.3 m/s to 18.3 m/s, *p* = 0.018), while control group subject did not (51.0 vs. 50.5, *p* = 0.581 and 15 m/s vs. 13.8 m/s, *p* = 0.893, respectively), 6MWT tended to improve in both groups.

**Conclusions:**

Our participants were able to perform correct balance proactive and reactive responses, indicating that older people are able to learn balance trunk and arm reactive responses during stationary bicycling. The pilot study shows that these improvements in balance proactive and reactive responses are generalized to performance-based measures of balance (BBS and Postural Sway measures).

**Supplementary Information:**

The online version contains supplementary material available at 10.1186/s12877-021-02015-1.

## Background

The rising proportion of older adults in the population and their associated morbidity is placing upward pressure on overall healthcare resources [1]. One serious health problem is falls, which are the leading cause of fatal and nonfatal injuries in this population. More than 30% of community-dwelling older people fall at least once a year, and this figure rises to about 50% among those aged 80 years and older [2–4]. A range of 20–30% of those who fall suffer acute injuries such as hip fractures and traumatic brain injuries that reduce mobility, independence, and even result in death [3, 5]. In 2015, in the US, the medical costs for older people’s falls was above $50 billion [6]. A critical role in preventing falls and preserving functional independence is played by balance control [5], specifically balance reactive responses evoked by an unexpected perturbation followed by loss of balance. Consequently, there is a need to develop technologies that will improve balance reactive responses to reduce falls in older people.

Ineffective balance reactive reactions are one of the major causes of falls in older community-dwelling adults [7]. Unexpected loss of balance such as a push, slip, or a trip trigger automatic postural responses which act to restore equilibrium [7–10]. These responses decelerate the center of mass (CoM) over the base of support (BoS) to prevent a fall from occurring [7], and these are specific to the size, type, and direction of the perturbation [7–10]. For example, fixed BoS strategies (feet remain in place) are used to restore balance by ankle, hip, trunk and arm movements during minor-to-moderate perturbation magnitudes. While at larger perturbation magnitudes, changes in BoS strategies (reactive stepping responses) are employed [7]. It was found that hip, trunk, and arm movements are also highly involved as a part of reactive balance reactions [8, 11–15], helping to decelerate the CoM movement over the BoS to recover balance at low, as well as high, perturbation magnitudes [7–9]. A recent laboratory study of 83 older people with varying histories of falls, who were exposed to a wide range of perturbation magnitudes, found that about 61% of these perturbed trials resulted in fixed BoS balance reactions without the need to recover balance by stepping [16]. Systematic reviews published recently found that perturbation training programs are effective in improving balance reactive responses, reducing fall incidence [11–13], and even reducing diverse risks such as wrist and hip fracture, traumatic brain injury, and the rate of falls [14, 15].

Perturbation training intervention programs are conducted by different mechatronic systems that provide external perturbations in standing and walking in various ways [11–15]. These devices are designed to specifically train the change of support (i.e., reactive stepping responses) in older people who are able to stand or walk independently without external support, i.e., holding handrails, for training sessions that usually last 20–45 min each [11–15]. In these perturbation training programs, reactive hip, trunk, and arm balance reactions are also trained [12, 14, 15]. However, many people are unable to participate in this type of training programs. These include: older people who are less able to walk independently on a treadmill without holding the handrails, high-risk people such as pre-frail and frail older people, older people suffering from a fear of falling, those in pre-walking rehabilitative phases, people with neurological disorders such as stroke or traumatic brain injury, and even children with cerebral palsy. Therefore, a better way to improve balance reactive response and to decrease the number of fall-related injuries in high-risk people may be to direct preventive efforts conducting perturbation exercises that do not require training while standing or walking. In order to match the perturbation training approach for these people, aiming to specifically train the reactive hip, trunk, and arm balance response, designing and developing a mechatronic system that provides balance training that includes perturbations while sitting can be valuable.

We were also inspired by the well-known health advantages gained after older people participated in bicycling training programs, such as improvement in cardiovascular parameters [17–19], increasing muscle power and endurance [19, 20], and improving executive function [18, 21] and quality of life [17]. Moreover, bicycling training improves gait parameters in older people [21], people with stroke [22], Parkinson’s [21], multiple sclerosis [23], and heart disease [19]. This is not surprising since pedaling and walking are lower-extremity rhythmic tasks with similar reflex modulation [24–26], and related neural circuitry may be operating in both tasks [27, 28]. We were also motivated by recent works that found that older people who bicycle outdoors regularly have better balance control than age-matched controls [29, 30], and the amount of outdoor bicycling was associated with the degree of balance control [31].

In this paper, we aimed to describe the design, development, building, and clinical applications of a novel mechatronic perturbation-based bicycle simulator system, the Perturbation Stationary Bicycle Robotic system (i.e., PerStBiRo). The PerStBiRo provides unannounced lateral external perturbations and self-induced perturbations during pedaling on an unstable “floating” surface (see details in the system description below). The PerStBiRo system specifically challenges trunk and arm proactive and reactive responses during bicycling in a safe sitting position that is suitable for older people at different levels of functioning. In addition, we describe the development of an artificial intelligence software that is able to identify the trainee’s balance reactive responses to control the motor of the PerStBiRo system. Finally, we report the results of a pilot RCT clinical trial that aimed to explore whether gait and balance function improved with PerStBiRo training. We hypothesized that participation in a recently developed PerStBiRo balance training program would show improvements in gait and balance function in older people.

## Methods

### Development of the perturbation stationary bicycle robotic system

#### System description

**Fig. 1:**
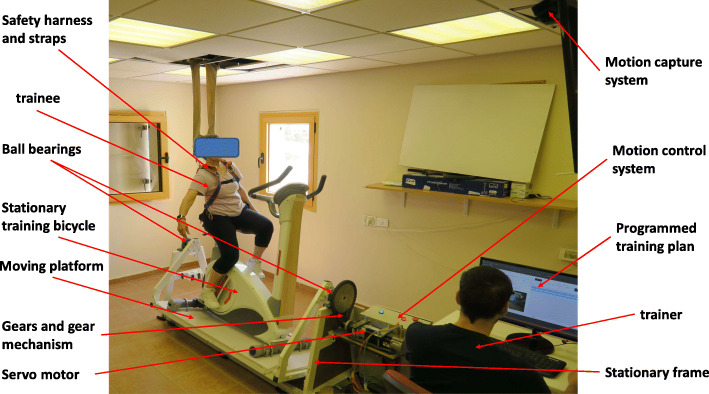
A Photo of the PerStBiRo system. The system is composed of a stationary training bicycle mounted on a moving platform, stationary frame, servo-motor, motion control system, gears and gear mechanism, two ball bearings, motion capture system, safety harness, and a trainer station (see the text for more details)

The Perturbation Stationary Bicycle Robotic system (PerStBiRo system) is a mechatronic device weighing 90 kg that provides lateral roll-angle (lateral tilt) balance perturbations during hands-on or hands-free bicycling in a safe environment (see Fig. 1). It is comprised of a stationary training bicycle (STB) that is mounted on a moving platform connected to a series of gears and a servo-motor. The gear ratios combine the servo-motor rotation with the platform rotation axis by two ball bearings, in the back and front of the platform, allowing platform rotation and, hence, balance perturbation tilts. The system provides a maximum right and left perturbation tilt angle of 20° (each side) with maximum acceleration and deceleration of 30 m/s^2 and a maximum velocity of 30 m/s. The motor that executes the perturbation tilts is controlled by a motion control system and a motion capture system (Microsoft Kinect™ system), which are both controlled by a main computer software program. The computer program is on the host PC that also serves as a user interface. By a program command, the motion control system directs the motor rotation based on a programmed training plan, which is usually that of a triangular motion profile for each perturbation (acceleration–deceleration). The computer software program allows the trainer to determine the training plan and to control all balance exercise parameters (maximum acceleration/deceleration, maximum velocity, and angle of perturbation, along with the number of right/left perturbation repetitions and the delay time between them). Once an unannounced balance perturbation is given, only when an appropriate balance reactive response is detected by the software i.e., based on the Microsoft Kinect™ camera’s, the moving platform tilt rotation (i.e., the perturbation) is stopped, and the motor returns the PerStBiRo system to its vertical position (i.e., its neutral/zero position) by motor counter-rotation. In addition, the program saves a file that logs the exercise completed for post-training analysis.

#### The PerStBiRo system to implement motor learning of balance control

Motor learning refers to the human internal processes associated with practice of a particular movement that leads to relatively permanent changes in the capability to respond [32]. The motor learning process improves with the practice of many repetitions of motor performance, leading to the person’s increased capability to produce the desired action. Varied practice in a random order has been found to result in better motor learning [33]. During our customized perturbation training program, the desired result is that the trainee will perform effective balance reactive responses with the trunk, head and arms to recover balance from a perturbation during hands-free bicycling. When the trainee performs effective balance reactive responses the PerStBiRo system, through its computer program and motion control system, is designed to progressively expose the trainee to repeated random unannounced balance perturbations at higher magnitudes (i.e., higher tilt angles). In this way the trainee gets real time feedback and thus may learn to perform an effective reactive balance responses. This intrinsic sensorimotor task cue, and the immediate real-time balance reactive response feedback, provides the learner (trainee) with an implicit cue to successful balance reactive response and provides the best possible motor learning implementation [34].

#### The main PerStBiRo system components

##### The servo-motor and gear mechanism

The moving-platform frame is connected from both ends (back and front) to two ball bearings. The front ball bearing connects to a gear mechanism and the servo-motor with a power rating of 2.97–3.38 kW. In the gear mechanism itself, there are two external gears (large and small) that are connected by a motor chain (see Supplementary Materials – Fig. 1 for the gear mechanism). Therefore, there are two internal transmissions with a gear ratio of approximately 1:5. The servo-motor is connected to a cylindrical component that is welded to the small external gear. The motor has a maximum speed of 3000 rpm and peak torque of 10.8 Nm.

##### The motion control system

**Fig. 2:**
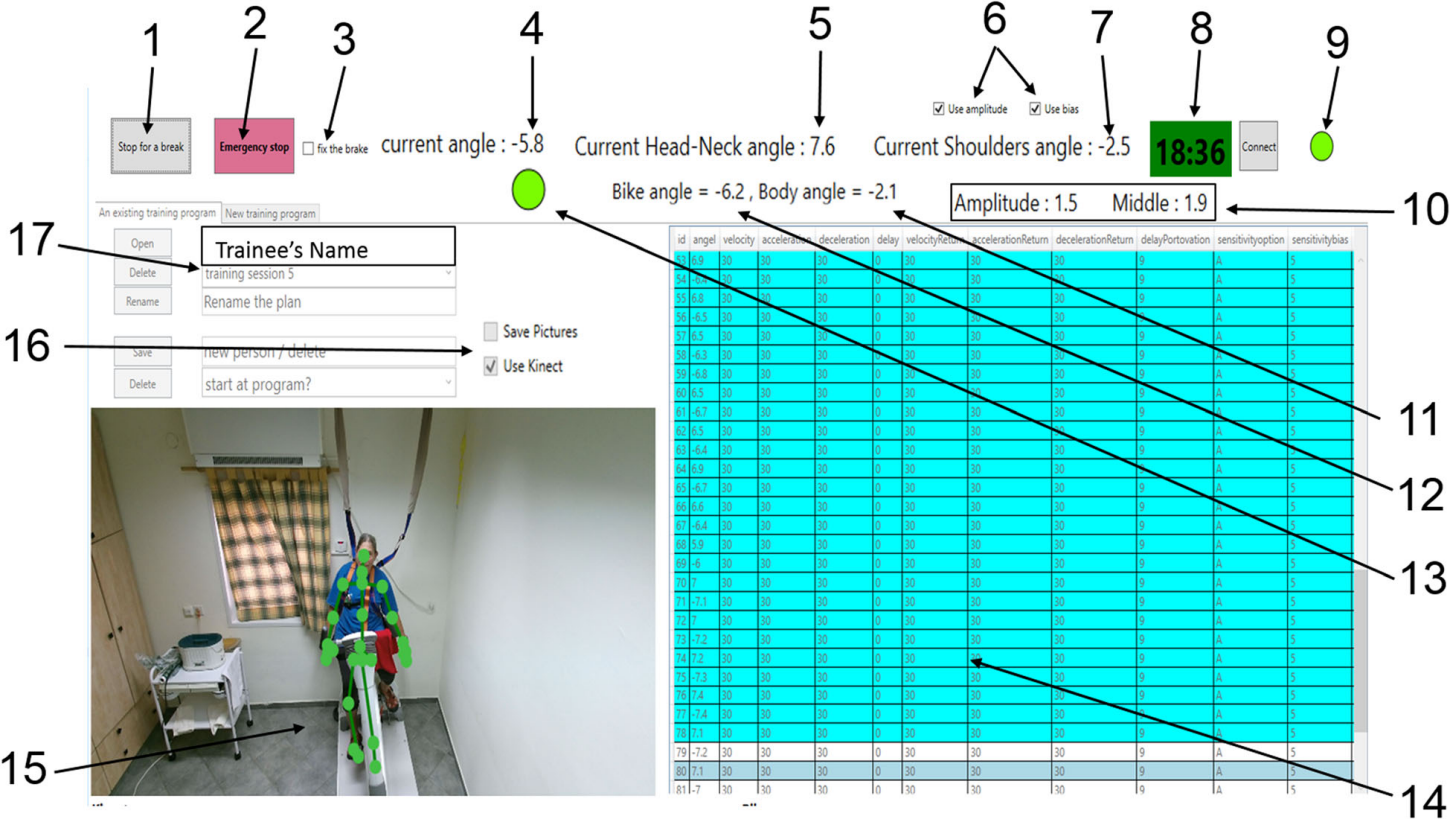
The “Run training-program” tab during a training session. 1 – Start/Pause/Continue options of the training program (button); **2** – Emergency stop (button); **3** – Fixes the moving platform in zero position (on/off button). An option for relieving a trainee who is having difficulty during training; **4** – Current STB angle; **5** – Current Head–Neck angle (α2); **6** – Using calibration parameters or not (button). An option for ignoring inaccurate calibration data for relieving a trainee who is having difficulty during training; **7** – Current Shoulder angle (α1); **8** – Training timer; **9** – Overall connectivity check of all components: green represents that everything is connected properly; **10** – Calibration parameters; **11** – Shoulder angle (α1) as absorbed during the last balance reactive response in the last perturbation; **12** – STB angle as absorbed during the last balance reactive response in the last perturbation; **13** – Green mark for identifying an effective balance response in the last perturbation or red mark for an ineffective response; **14** – Ongoing customized training program (blue lines represent the past perturbation and write in the next one); **15** – Real-time trainee stick figure; **16** – Control and operation of the Microsoft Kinect™ system (on/off button); **17** – Trainee’s name, training program’s name, viewing the training history option

The motion control system is based on the motion controller (Supplementary Materials – Table 1). The computer program uses the motion control system to communicate with the servo-motor, which in turn executes the tilting perturbation of the moving platform. From the computer program, the motion controller receives the required information about the direction, tilt angle, maximum velocity, and maximum acceleration/deceleration. The motion controller has an internal motion profile generator that produces a triangular velocity profile. The moving platform/STB accelerates to generate the required tilting perturbation and then decelerates to zero velocity in both situations, whether or not a balance reactive response is detected (see Supplementary Materials – Fig. 2 for the motion control system interactions).

##### The Microsoft Kinect™ system

The Microsoft Kinect™ camera is mounted at a 45° horizon angle at a height of 2.8 m and 3 m in front of the trainee’s sitting position for the best motion capture of the trunk and arms’ reactions, without being hidden from the STB’s handlebars. The Microsoft Kinect™ system captures the body posture in real time and allows implementation of the upper-body balance reactive responses to be monitored and increased. The Microsoft Kinect™ sensors collect the trainee’s body movements with respect to the current PerStBiRo system state and analyzes their responses to ongoing events. First, by calibration phase, we identify the effective balance response threshold for each trainee. Second, following a perturbation during a training session, provides a customized intrinsic sensorimotor cue for the trainee’s effective reactive responses, by stopping the perturbation immediately and returning the PerStBiRo system to its vertical position [33, 34]. In addition, the Microsoft Kinect™ system allows the trainer real-time tracking of the balance reactive responses throughout a current training session and to monitor progress throughout the training sessions. We found high correlations (*r* = 0.75–0.78, *p* = 0.04) between Microsoft Kinect™ vs. APAS 3D motion capture system for the leg balance recovery responses for 8 healthy young adults who were exposed to unexpected perturbations during standing and walking (*r* = 0.75–0.78, *p* = 0.04) [35]. Also, no systematic bias was evident in the Bland and Altman graphs (details in [35]). Thus, using the Microsoft Kinect™ system provides comparable data to a video-based 3D-motion analysis system when assessing balance reactive responses in the clinic.

We used the upper-body joints that the Microsoft Kinect™ interface provides because the balance, trunk, and arm movements are the training target. We calculated several angles (α1-α6) that we considered in our computer program: **α1)** Shoulder angle: The angle of the line between the trainee’s two shoulders and the ground.; **α2)** Head–Neck angle: The angle of the line from the trainee’s head to neck and the line vertical to the ground; **α3)** Head–ShoulderBase angle: The angle of the line from the trainee’s head to the SpineShoulder joint and the line vertical to the ground; **α4)** Head–Calculated Shoulder Center angle: (Definition: CalculatedShoulderCenter lies in the middle of the two shoulder joints given by the Kinect.) The angle between the line from the head to the CalculatedShoulderCenter of the trainee and the line vertical to the ground; **α5)** Left elbow angle: The angle between the line from the ElbowLeft to the ShoulderLeft joints of the trainee and the line vertical to the ground; **α6)** Right elbow angle: The angle between the line from the ElbowRight to the ShoulderRight joints of the trainee and the line vertical to the ground. We used angles α1 and α2 in the real-time training process to identify the trainee’s body position with respect to the STB in order to compute the moment an effective balance reactive response was shown and tilting back of the STB. Angles α3–α6 were not used during the real-time training process, but are shown in post-training graphs for advanced post-training analysis. Monitoring the trainee’s balance responses over time can indicate the implementation of skill acquisition and the motor learning progress of the balance upper-body reactive responses. The data of angles α3–α6 reveals to the trainer all the information about the balance reactions. For example, the arm reactions (α5 and α6) that are part of the training target, shows occasionally less accurate and noisy information; thus, the calculation is not based on these angles. However, it can be helpful in understanding the entire response to perturbations. We logged all body part locations in each frame taken by the Microsoft Kinect™ system for post-training calculations of these angles.

##### Software and user Interface

The computer program serves as the user interface and runs on the host PC. The program’s application is of a Windows format and was designed to have a user-friendly interface containing three tabs:

**Run training-program tab**

In this main tab (see Fig. 2 for details), the trainer selects a suitable perturbation program and runs it. The trainee’s training history is opened so that the trainer can make better treatment progress decisions. The desired training program is chosen from a pool of programs created to date. The operation of the Microsoft Kinect™ system is controlled with this tab. The emergency stop button is also here. In addition, all perturbation parameters, as well as the last balance reaction parameters and the real-time STB angle, trainee’s shoulder line angles (α1), and head–neck angle (α2) are displayed on the host-PC screen (Fig. 2, numbers 4, 5, 7, 11, 12). Thus, the trainer can compare the programmed perturbation angle with the actual angle (once an effective balance response is detected) and monitor the patient’s balance reactive responses after perturbations along the training session. For receiving better sensorimotor biofeedback and allowing a better fit to the subject’s ability, the balance reactive response threshold parameters, based on the automatic calibration phase, is the only set of parameters the trainer can calibrate in real time while running a training program (Fig. 2, number 6). All the features presented in Fig. 2 are to help the trainer with real-time monitoring of the balance reactive response and to give options for adapting the difficulty of a selected training program to each trainee individually.

**Creating training program tab**

This tab enables the creation of a new customized training program. In the setting process, each perturbation is programmed separately and added in chronological order to the list of perturbations. For each perturbation, the trainer sets the maximal desired values of the motion profile parameters, as long as a balance reactive response is not detected by the Microsoft Kinect™ system (acceleration, deceleration, velocity, and perturbation angle). The delay time between each two consecutive perturbations, the tilting direction, and the number of perturbations during a single training are also set by this tab.

**Saving data tab**

This is a pop-up exiting window tab that verifies how data are stored. The program gives the user either the option to save or discard the video frames taken by the Microsoft Kinect™ system. In both cases, the program saves an output Excel file that contains all the motion parameters of the moving platform and the trainee’s body balance proactive and reactive balance responses.

#### Safety harness

Safety is an extremely important issue since, in this training, we apply unexpected perturbations that may cause older people to fall off of the PerStBiRo system. A safety harness is suspended from the ceiling by two ropes above the trainee (Fig. 1). In case the trainee fails to recover and falls from his/her seat, the safety harness will arrest the fall before the trainee can fall from the bicycle seat. The harness is slightly loose to be safe, but not restrict balance response.

### Types of perturbations

The PerStBiRo system provides right and left tilting balance perturbations that aim to challenge the hip, trunk, and upper body balance reactive reactions. The tilting perturbation axis crosses along the STB and passes under the trainee’s seat position, while the trainee’s CoM that is located in the pelvis is above the bicycle seat. Therefore, when tilting the trainee’s CoM aside rapidly, the trainee is forced to decelerate the CoM by responding reactively with hip, trunk, and arms balance reactions during pedaling. Balance perturbations are provided in two forms: 1) machine-induced unannounced external perturbations and 2) self-induced internal perturbations during hands-free cycling. The external perturbations are controlled programmed machine-induced and are ranged from low to high magnitude (0°–20° for each side). They can be programmed expectedly as a block perturbation training (fixed time, order, and magnitude) which is under proactive balance control training, or be given unexpectedly as random perturbation training (in onset, magnitude, direction, and time interval) which evoke fast upper-body reactive balance responses (i.e., trunk, hip, head, and arm reactive movements). The internal self-induced perturbations are provided by the unfixed and unstable mode of the PerStBiRo system. Its moving platform has a fixed mode or unfixed “floating” mode, where it acts like a surfboard floating on the water and is subjected to the forces exerted on it by the trainee during pedaling. These situations fall under proactive balance control training. This unstable mode is programmed for the time interval between the external parturitions.

### PerStBiRo system communication and activation

The PerStBiRo system has a programming mode (editing mode) and working mode. The safety micro-switch and pin determine its mode. When the safety pin is closed (pressed micro-switch), the servo-motor is off, and the trainer can edit or create a new training program by the user interface, but cannot start running it. When the safety pin is open (undressed micro-switch), the servo-motor receives electric current, and the user may start the training program, but cannot change data on it. Thus, for running a training program, the physical therapist (trainer) first creates a customized perturbation training program with the user interface (programming mode). Second, in working mode, the computer program runs the training program by utilizing the motion control system and the Microsoft Kinect™ system, both controlled by the computer program. The Microsoft Kinect™ system transmits information about the trainee’s movements, while the motion control system is responsible for receiving information and delivering commands to the servo-motor. For executing a perturbation command, the motion control system activates the servo-motor, then the moving platform (with the STB and the trainee) tilts laterally, and the programmed perturbation is executed. in case the software detects an appropriate trunk and arms balance reactive response i.e., based on the Microsoft Kinect™ cameras, the perturbation is stopped, and returns to its vertical position. In addition, the trainee’s heart rate is monitored using a smartwatch (TomTom Runner Cardio [see details in [36]]) under the trainer’s control all along the program training. After the training program, the physical therapist may review the trainee’s balance reactive movement graphs, and the data collected in order to determine how to proceed in the next training session. See Supplementary Materials Fig. 3 for details of the system communication flow chart.

### The Microsoft Kinect™ system function during the training

**Fig. 3:**
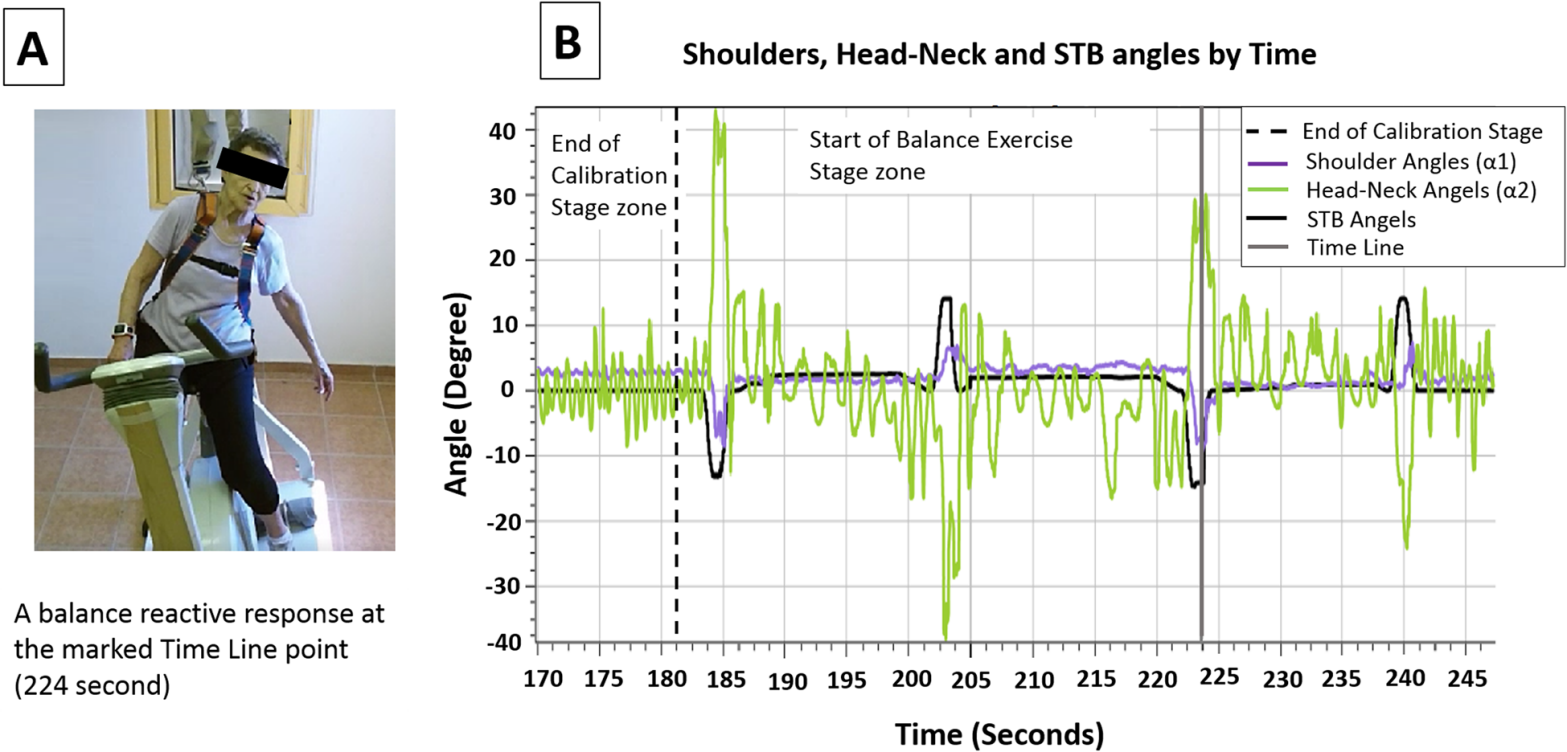
Example of the Kinect-system function during random-unexpected perturbation training process of an 82-year-old advanced level trainee, focusing on a reactive balance response following a 20° left unannounced tilting perturbation. Figure 3**a** represents the balance reactive response detection from the Microsoft Kinect point of view. At the end of the calibration stage (Fig. 3**b**, dashed vertical black line), the computer program and the Kinect system automatically customize the PerStBiRo system to the current trainee by calculating both: 1) the individual’s upper body sway amplitude, and 2) the trainee–stationary training bicycle (STB) zero point (i.e., vertical position). The customization process – α1 and α2 angles (presented in Fig. **b** – green and purple lines) are recorded separately for 90 s during the second part of the calibration stage, and at the end of this stage, the individual upper body sway amplitude and the trainee–STB zero point are calculated for both angles (α1 and α2). Then the angles that show more stability and less noisy parameters are automatically selected as the angle on which the software relies to give real-time sensorimotor feedback for an effective balance reaction by returning the STB to its vertical position. After this, the balance exercise stage begins, and the trainee is exposed to a variety of repeated random unexpected perturbations (Fig. 3**b**, humps in the horizontal black lines). When a 20° left perturbation is executed (the gray time-line line in Fig. 3**b**), the computer program checks the difference between the participant’s angle (Fig. 3**b** – purple line α1 or green line α2) and the training bicycle’s angle (Fig. 3**b** – black line) and considers the body amplitude of the participant and the trainee–STB zero point to see if there has been a significance balance reactive recovery response rather than a regular paddling movement. In this case (Fig. 3**b** – where the gray time-line is), the PerStBiRo system detected a reactive balance reaction (**a**) as the stationary training bicycle tilted only 15° (Fig. 3**b** – black line) out of the 20° that was programmed in the training plan

Figure 3 presents a short sample of the Microsoft Kinect™ function during a training process. Each training session usually lasts 20 min, there are two stages: 1) the calibration stage (the first 3 min) and 2) the balance exercise stage (17 min). 1) The calibration stage is for minimizing errors in our Microsoft Kinect™ system’s calculations and for automatically customizing the PerStBiRo system to the trainee who is currently using it. It consists of two parts: A) The trainee’s adaptation phase – 90 s of warning-up slow pedaling to allow the trainee to ease into a comfortable position. In this phase, the computer program does not make any reference point calculations due to the noisy data that was gathered by the Microsoft Kinect™ system before the trainee fixed his seat position. B) Measuring and calculating the individual upper body sway amplitude (the body-sway base noise) and the trainee-STB zero point – 90 s of self-paced pedaling while the Microsoft Kinect™ system provides data to the computer program for calculating angles α1 and α2, as described previously (see Fig. 3). At the end of the calibration stage, the computer program calculates customized reference angles that will be used later to determine whether the trainee’s balance reactive responses were effective or not. Based on the identified minimal body sway amplitude, the computer program detects if the trainee responded to a given perturbation or whether their current body angle was a part of natural movement during pedaling (i.e., into the body-sway-base-noise range). Thus, first, the computer program records the data of the α1 and α2 angles during the 90-s of calibrated self-paced pedaling. Second, it calculates separately for each angle (α1 and α2) the upper-body sway amplitude and the trainee’s STB zero point. We approximate the amplitude of each angle (α1 and α2) by using this formula: {Max (angles) – Min (angles)}/2, where angles are the list of all the angles that were recorded. The trainee–STB zero point is the angle at which the trainee is naturally sitting on the STB during pedaling, and this is necessary because often older people naturally tend to lean a few degrees to either the left or right side. Third, based on the least “noisy” angle during the calibration stage (α1 or α2), the computer program automatically selects to use its upper body sway amplitude and the trainee–STB zero point to determine whether the movement that the trainee has made is a reaction to the perturbation or if it was just the effect of pedaling. 2). The balance exercise stage (about 17 min) contains block and expected or random and unexpected balance perturbations (Fig. 3). When a new perturbation is executed, the computer program checks the difference between the trainee’s angle (calculated by the chosen formula, α1 or α2, following the calibration stage, Fig. 3) and the STB’s angle (computed using the trainee–STB zero point of α1 or α2 and the trainee as an anchor for this calculation) and takes into account the body amplitude of the trainee to see if there has been a significant movement other than a pedaling movement.

We programmed two options for separating a balance reactive response to a normal pedaling movement following a perturbation: Option A – checking if the STB is leaning in the opposite direction to the body movement, above a threshold angle. The threshold is the summation of the trainee’s body amplitude and a programmed predetermined bias. This additional selected bias requires the trainee for a larger distinct balance reactive response to recover their balance for passing above the response threshold and stopping the perturbation by turning the STB back to its neutral position. This option deals with a trainee who exhibits large body amplitudes during the exercise session versus the calibration phase. We denote this summation threshold “diffAllowed”. If the STB and the trainee’s body are leaning in opposite directions, the computer program checks if the current body angle is larger than [trainee–STB zero point + (diffAllowed /2)]. If so, then the trainee performed both, moved in the opposite direction of the STB, and passed its trainee–STB zero point. Next, the command to stop the perturbation and return the STB comes out. Option B - checking if the trainee is leaning in the opposite direction of the STB, regardless of the trainee–STB zero point.

### Exploring balance proactive and reactive responses

Here, we present results of a proof of concept study to explore whether the PerStBiRo software was able to identify balance reactive responses. Two people (i.e., a young and an 86-year-old male) were exposed to unannounced lateral balance perturbations while sitting on the PerStBiRo system. The tilting perturbations evoked balance reactive trunk, head, and arm movements always in the opposite direction of the perturbation to quickly move the upper body’s CoM toward the base of support provided by the STB seat. The ability of both participants to perform upper body balance reactive responses was improved within one training session.

**Fig. 4:**
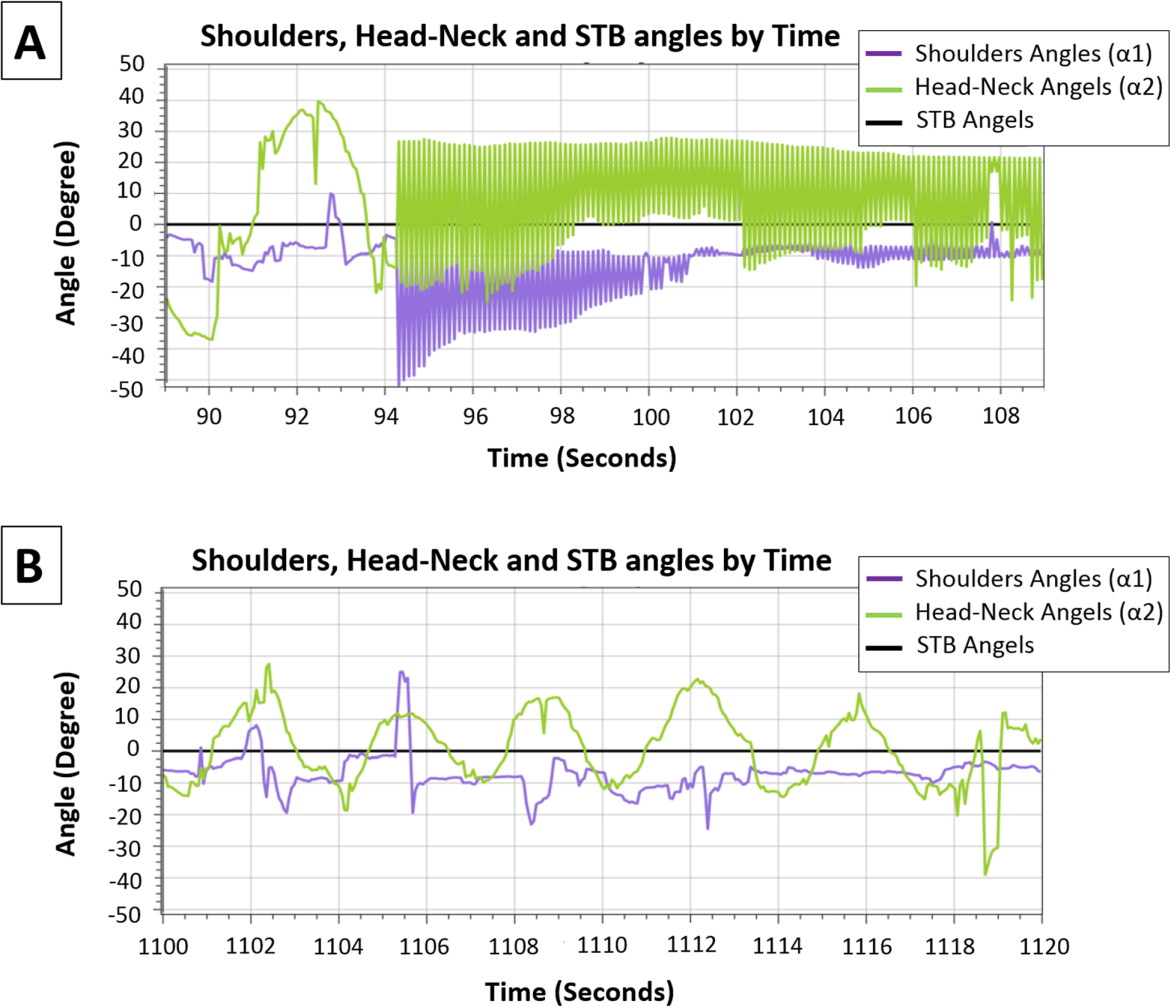
The PerStBiRo system’s ability to monitor and identify skill acquisition of hands-free pedaling during training session. Fig. 4**a** –A sample of about 20 s, note that the 86 years old trainee released his hands from the handlebars (about 94 s of the training session), which was immediately accompanied with upper body instability, i.e., shoulder and head angle instability, during pedaling [purple (α1) and green (α2) line]. Figure 4**b** – the end of the training session (1100–1120 s of the training session). A sample that represents better upper body stability (i.e., lower amplitudes) during pedaling [purple (α1) and green (α2) lines]

**Fig. 5:**
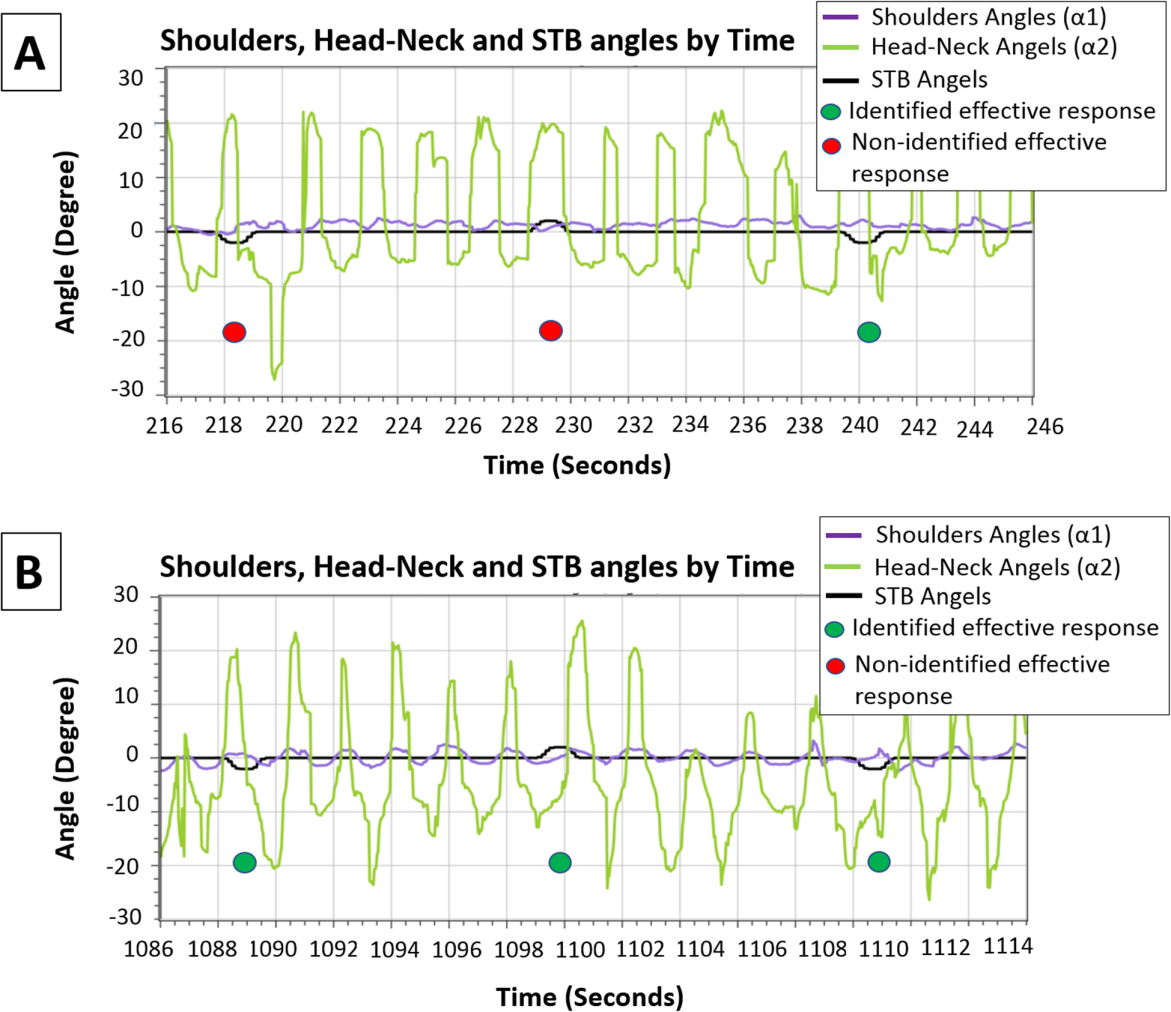
An example of the ability of the PerStBiRo system to detect (green and red circles) and train a reactive balance response during announced perturbations. Perturbations of 2.5° (little humps in the black lines). Figure 5**a** –A sample of 30 s that represents the 86 years old trainee’s inability at the beginning of the training to actively respond to announced external perturbations at low magnitudes, accelerations, and velocities that were provided as right–left 2.5° unannounced tilt [shoulder purple line (α1) shows mismatch between responses and perturbations (humps in black line)[. Figure 5**b** –A sample of 30 s that represents the trainee’s ability to consistently react at the end of that training session [purple line (α1) reacts in the opposite direction and related to the black line perturbations]. In cases of low unannounced perturbation magnitudes (2.5° tilt), the PerStBiRo system identified an effective balance reaction, but does not return the PerStBiRo system back to its vertical position; thus. no sensorimotor feedback is given

The skill acquisition and motor learning of the 86-year-old male during several training sessions is demonstrated in Fig. 4 and Fig. 5. This participant had reported several falls in the past 6 months, expressed a fear of falling, and self-reported a reduction in his level of physical activity. His upper-body balance reactive responses are presented by the shoulder line (α1) and head–neck (α2) angles. These angles were found to be the best parameters to distinguish the presence of an upper-body balance reactive response.

In Fig. 4 which was the first training session, the participant pedaled with, and then without, holding the bicycle handlebars (i.e., hands-free) for 20 min without perturbation. Exercises in this session represent little actual challenge to the balance control system. The goal of this training session was mainly directed towards a cognitive understanding of the exercises, and an improvement in self-confidence for training at higher levels. Figure 4 shows the PerStBiRo software’s ability to monitor and identify the process of the participant’s sensorimotor adaptation to hands-fee pedaling. The first phase is to release the hands from the handlebar about 94 s into the training, which causes massive and noisy upper-body movements (Fig. 4a). The second skill acquisition phase is demonstrated by very organized and reduced upper-body movements (Fig. 4b) by the end of the first session. This represent an improved balance control ability.

After two training sessions i.e., the third training session (Fig. 5), the participant was exposed to low magnitude announced perturbations (2.5°) during hands-free pedaling. Figure 5 shows that, at the beginning of the training session, the PerStBiRo software was unable to accurately identify balance reactive responses (Fig. 5a), although the participant proactively and reactively responded to balance these perturbation responses. Later in the same training session, the PerStBiRo software able to detect proactive and reactive balance responses and, thus, control the PerStBiRo motors.

### Post-training kinematic data analysis

Observing and analyzing a trainee’s performance of past training sessions can be useful for making a clinical decision regarding the progress of rehabilitation, and for indicating skill acquisition and motor learning progress of the balance trunk, head, and arm reactive responses. Additional software enables the trainer to observe the kinematic data of a specific trainee in a specific training session. It presents kinematic graphs and a moveable timeline that allows the trainer to observe the α1–α6 angles and the Microsoft Kinect™ system’ body stick figure at every timestamp, compared to the STB’s angle position at that timestamp.

### Pilot clinical trial

#### Study population

In a small randomized controlled pilot study, we recruited 13 community-dwelling older people from retirement housing (see characteristics in Table 1). Eligibility criteria were: 70 years of age or older, ability to walk independently, a Mini-Mental Score higher than 24, no severe focal muscle weakness or blindness, no known neurological disorders, and no metastatic cancer. All subjects provided a medical waiver signed by their primary care physician clearing them to participate in moderate physical exercise. The study was approved by the Helsinki committee of Barzilai University Medical Center, Ashkelon, Israel (BARZI0104). All subjects signed an informed consent statement.

**Table 1:** The clinical pilot study participant characteristics. Values are medians (Min, Max). Because of the small sample size, group comparisons were based on the Mann–Whitney *U* test and Chi-square for sex

Characteristics	PerStBiRo Training (*n* = 7)	Stationary Bicycling (*n* = 6)	***P*** -value
Age (year)	83 (73–86)	81.5 (74–86)	0.84
% Female (No.)	57 (4)	83 (5)	0.30
Height (centimeters)	160 (150–180)	155 (151–167)	0.53
Weight (kilograms)	66.5 (60–107)	65.5 (52–82)	0.53
Mini-Mental score	29 (26–30)	28 (28–29)	0.23
No. diagnosed diseases	3 (0–4)	2 (1–4)	1
No. drugs/day	2 (0–8)	4 (2–9)	0.45
No. falls /last year	0 (0,2)	0 (0,4)	1
Fall Efficacy Scale International score (FES-I)	23 (19, 35)	33 (27, 43)	0.02
Berg Balance test score	54 (49, 56)	51 (47, 55)	0.23
Postural sway velocity (mm/sec)	20.3 (9.0, 28.2)	15.0 (9.9, 18.1)	0.05
Six-Minute Walk Test (meter)	450 (323, 480)	376 (262, 393)	0.18

### Study design

After eligibility and baseline assessments, 13 older people were randomly allocated to one of two intervention groups 1) PerStBiRo training or 2) stationary bicycling. The random allocation was made by an investigator who was not involved in the assessments using computer random allocation software (Random Allocation software version 1.1, Isfahan, Iran). Postural stability, along with performance-based balance and gait functions were tested before and after the training period by a blinded investigator. All assessment sessions were performed at the same time of day, and in the same order.

### Training programs

The PerStBiRo provided controlled unannounced perturbations and self-induced medio-lateral titling perturbations during the stationary bicycling for the intervention group. The control group trained on the same system, but without perturbations. Both groups participated in 20 training sessions, twice a week for 10 weeks. Each session lasted for 20 min and included three parts: 1) warm-up – 3 min of self-paced pedaling with the same bicycle resistance for both groups that gradually increased, 2) main exercises – 15 min of internal and external perturbation training during hands-free stationary bicycling or bicycle riding on the PerStBiRo system in its fixed mode while holding the handlebar with no internal or external perturbations, and 3) cool down – 2 min of self-paced pedaling. In both groups, the trainee was instructed to “ride the bicycle at your preferred pace and try to stabilize yourself. This is a submaximal exercise, so try to stay within the heart rate range set in the pulse watch (60%–80% of maximal pulse).”

The difficulty/magnitude of the perturbation training level was adjusted according to each participant’s ability, starting from the lowest level at the first training session (practice hands-free pedaling). If the PerStBiRo software detected effective balance recovery responses of the trainee in most unannounced perturbations during a specific training session, a higher level of perturbations was introduced in the next session. If not, the same level of perturbations was used again until the participant could successfully perform balance recovery responses during the entire training session. The participants were encouraged to perform exercises with no or minimal external support. However, assistance and support were provided for participants who felt unsafe in the initial phase of the training.

### Assessments

Before and after 20 training sessions, we measured postural stability in upright standing. The participants were instructed to stand barefoot as still as possible on a force platform in a standardized stance, their feet close together with their hands crossed behind their back [29]. Five 30-s eyes-closed (EC) assessments were conducted for each participant. CoP (Center of Pressure) and ground reaction force data were collected at a frequency of 100 Hz with a single Kistler 9287 force platform (Kistler Instrument Corp., Winterthur, Switzerland). Balance control was evaluated using the traditional measures of CoP postural sway (e.g., ML sway range, AP sway range, mean sway velocity, mean sway area). The data were extracted from the CoP trajectories using a code written in MatLab (Math Works Inc., Cambridge, MA, USA). We also performed the Berg Balance Scale (BBS), a 14-item performance-based measure of functional balance [37] and the Six-Minute Walk Test (6MWT) measuring the maximum distance that a person could walk in 6 min [38]. We calculated Effect Size (ES) for each parameter using this formula: ES = [(Δ PerStBiRo training – Δ Stationary bicycling training)/(mean SD of both groups)].

## Results

### Pilot clinical trial

The PerStBiRo perturbation training resulted in a significant decrease in CoP sway velocity (20.3 m/s (19, 35) to 18.3 m/s (8.4, 26.1), *p* = 0.018, ES = 0.46) (data presented as Median (Min, Max)), and an increase in the BBS (54 (49, 56) to 56 (53, 56), *p* = 0.026, ES = 0.59), while control group subject did not (15.0 m/s (9.9, 18.1) to 13.8 m/s (9.4, 18.0), *p* = 0.893, and 51 (47, 55) vs. 50.5 (49, 55), *p* = 0.581, respectively). The 6MWT tended to improve in both training groups.

With respect to side effects and adverse events, during the exercise training program, muscle soreness was experienced by some subjects, especially in the early stage of the training, and one subject from the PerStBiRo training, reported that his chronic back pain had slightly increased for the next few days after the 16th training session. These effects were managed by adjusting the training intensity, and the symptoms disappeared during training.

## Discussion

We found that the PerStBiRo system can evoke upper-body balance proactive and reactive responses in older people. We also demonstrated that using the Microsoft Kinect^KM^ camera, the software that was specifically developed for this project was able to detect upper-body balance proactive and reactive responses in an older person and enhance the motor learning process of their balance proactive and reactive responses. Our pilot RCT suggests that 3 months of PerStBiRo training can improve balance function in a group of older people.

### Perturbation progress and clinical applications

Based on the PerStBiRo system’s features and our pilot results, and with respect to principals of motor learning, strength endurance, and especially balance control, we suggest a gradual increase in the difficulty of a PerStBiRo training program:

1. External support. It was previously found that holding onto the handlebars can significantly reduce the postural responses [39]. Thus, the main goal of this perturbation program was to practice hands-free cycling. We found that even an 86-year-old who suffered from balance impairments could adapt to this challenging situation.
2. Progressive overload, gradually increasing the perturbation magnitudes (i.e., increasing the tilt, velocity, and accelerations of the moving platform). For beginner trainees, we suggest starting with low perturbation magnitudes, but to increase the magnitudes to trigger balance reactive responses based on the trainee’s abilities and to improve balance reactive skills.
3. Random versus block training. The type of training method should be varied according to the trainee’s ability. For a low-functioning patient or frail older people, a low magnitude of perturbations that are expected and in block order (i.e., fixed time interval, direction, and magnitude) is recommended; thus, the trainee is aware of the direction of the perturbations and the timing (right or left–right, with a signal 5 s ahead of the perturbation). In contrast, an advanced trainee should be exposed to moderate-to-high unannounced perturbation magnitudes in random order (in onset, magnitude, and direction). Varied practice in a random order was found to better improve motor learning [33, 34, 40, 41].
4. Augmented feedback, implicit versus explicit. The external cue type also should change from no cue at all in the first few sessions to an external sensorimotor cue that leads to improving intrinsic sensorimotor feedback. Once an unannounced balance perturbation is given, when an appropriate balance reactive response is detected, the PerStBiRo returns to its vertical position. This intrinsic task feedback provides the learner (trainee) with an implicit cue for successful balance response, and provides the best possible motor learning implementation [33, 34].
5. Overload. The pedaling intensity should also increase along the training process, with the aim of improving lower limb muscle endurance and power as well.
6. Repetitions. Unannounced perturbations will be provided so that the participant will explore the best way to recover. The number of repetitions will be increased during the training sessions.

### Training session duration and training period

According to perturbation training paradigms in standing or walking, a feasible program for frail older people includes low perturbation magnitudes and requires longer training periods for significant improvement of reactive balance responses and reduction in fall incidence [12]. Since the PerStBiRo system focuses on improving upper-body balance reactive responses in a sitting position, it is suggested to include 20 training sessions over 3 months, applied at lower magnitudes of perturbation training during standing or walking at magnitudes that usually evoke upper-body balance responses. To adapt the treatment to the population of frail older people and for the time frame of physiotherapy treatments, we suggest that each session last for 20 min, that includes 3-min self-paced warm-up pedaling (which is also the time frame for calibration) and 17 min of perturbation training.

### Target population

Generally, the target population consists of older people who cannot handle with external perturbation training in standing and walking without holding the handrails (e.g., pre-frail or frail older people, old people who suffer from a fear of falling).

### Pilot study results

Our pilot RCT study results suggest, with caution, that unannounced external perturbation training while sitting can improve standing balance control, as well as performance-based balance in older people. The postural sway parameters and BBS score have been shown in the past to be associated with falls [42–44], and even to predict injury from falls [45]. These show that the benefits of unannounced perturbations during sitting can be generalized to other aspects of balance function (BBS score and postural sway measures). These findings suggest that the central nervous system makes adaptive improvement to stability as a result of trial-and-error perturbation practice. Not surprisingly, the 6MWT performance improved in both groups, further suggesting that, in regard to endurance, both training programs are effective.

### Strengths

This is a novel intervention method of a technology that provides self-induced and unannounced perturbations during stationary bicycle riding, which is designed to improve balance function during standing and walking among older people. It touches on an important point in the field of fall prevention, as well as rehabilitation and motor learning principles – the ability to transfer balance recovery reactions that are acquired in a sitting position into a target context of balance control performances in standing and walking. All these components provide the best possible motor learning implementation of reactive balance response in a sitting position and allow these exercises to be customized according to each subject’s ability. The exercises are challenging, but never dangerous. Our training is designed similar in time to conventional physical therapy treatments so that, in future clinical applications, it can be fit in a standard physical therapy session.

### Weaknesses

The PerStBiRo aims to improve balance control and balance reactive components by tilting perturbations. However, these perturbations during bicycle riding may not be similar enough to balance loss situations that cause real-life falls among older people; thus, they may not be specific. Because of the lack of specificity in this model, we may find no effects of the intervention on fall reduction. Our RCT had a pilot character and did not include quantitative measures of reactive balance in standing and walking. This program is less challenging than the Perturbation Balance Training in walking and standing, but our pilot results suggest that it is still possible for relatively healthy older people to improve balance skills. It is still unknown if PerStBiRo training could be beneficial for older persons who have many diseases and health conditions. Lastly, since the PerStBiRo is a challenging training approach, there is a risk that older people will stop participating in the program.

## Conclusions

This paper describes the PerStBiRo System that provides programmed controlled small-to-high unannounced lateral balance perturbations during stationary bicycling. We showed that the software program designed specifically for the PerStBiRo System was able to identify and analyze trainees’ balance proactive and reactive performance using the Microsoft Kinect™ system. We also showed that, in a relatively short period of training time (i.e., during one training session), an 86-year-old participant was able to perform correct balance proactive and reactive responses, indicating that he was able to learn upper-body reactive balance responses. In addition, we found in a small RCT that balance function of independent relatively healthy older adults who did not bicycle regularly and who participated in a 3-month PerStBiRo training program significantly improved their balance function. Future larger randomized controlled study should investigate whether older people who do not bicycle regularly can improve their balance control and balance reactive responses. Furthermore, a randomized controlled study should investigate whether patient populations who are unable to perform perturbation training that includes treadmill walking (e.g., pre-frail and frail older people, people with stroke, children with cerebral palsy, or those with traumatic brain injury and partial spinal cord injuries) can improve their balance recovery responses, function in standing and walking, and reduce their rate of falls.

## Supplementary Information

**Additional file 1: Fig. S1.** Gear mechanism

**Additional file 2: Table S1.** details of each hardware component not manufactured in-house

**Additional file 3: Fig. S2.** The Motion control system interactions

**Additional file 4: Fig. S3.** System communication flow chart.

## Data Availability

The datasets used and/or analyzed during the current study available from the corresponding author on reasonable request.

## Abbreviations

PerStBiRo: Perturbation stationary bicycle robotic system
STB: Stationary training bicycle
CoP: Center of pressure
ES: Effect size
BoS: Base of support
CoM: Center of mass

## References

[1] National Institute on Aging (2007). Growing older in America: the health and retirement study.

[2] Bergen G, Stevens MR, Burns ER. Falls and fall injuries among adults aged ≥65 years — United States, 2014. Mmwr Morb Mortal Wkly Rep. 2016;65:993–8. 10.15585/mmwr.mm6537a2 10.15585/mmwr.mm6537a2 27656914

[3] Centers for Disease Control and Prevention, National Center for Injury Prevention and Control. Web–based Injury Statistics Query and Reporting System (WISQARS) [online]. https://www.cdc.gov/injury/wisqars/index.html. Accessed 5 Aug 2016.

[4] Rubenstein LZ (2006). Falls in older people: epidemiology, risk factors and strategies for prevention. Age Ageing.

[5] Stevens JA (2005). Falls among older adults—risk factors and prevention strategies. J Saf Res.

[6] Florence CS, Bergen G, Atherly A, Burns ER, Stevens JA, Drake C. Medical costs of fatal and nonfatal falls in older adults. J Am Geriatr Soc. 2018 March. 10.1111/jgs.15304. 10.1111/jgs.15304 PMC6089380 29512120

[7] Maki BE, McIlroy WE (1997). The role of limb movements in maintaining upright stance: the "change-in-support" strategy. Phys Ther.

[8] Nashner LM (1976). Adapting reflexes controlling the human posture. Exp Brain Res.

[9] Nashner LM (1980). Balance adjustments of humans perturbed while walking. J Neurophys.

[10] Nashner LM (1977). Fixed patterns of rapid postural responses among leg muscles during stance. Exp Brain Res.

[11] McCrum C, Gerards MHG, Karamanidis K, Zijlstra W, Meijer K (2017). A systematic review of gait perturbation paradigms for improving reactive stepping responses and falls risk among healthy older adults. Eur Rev Aging Phys Act.

[12] Gerards MHG, McCrum C, Mansfield A, Meijer K (2017). Perturbation-based balance training for falls reduction among older adults: current evidence and implications for clinical practice. Geriatr Gerontol Int.

[13] Sherrington C, Whitney JC, Lord SR, Herbert RD, Cumming RG, Close JC (2008). Effective exercise for the prevention of falls: a systematic review and meta-analysis. J Am Geriatr Soc.

[14] Mansfield A, Wong JS, Bryce J, Knorr S, Patterson KK (2015). Does perturbation-based balance training prevent falls? Systematic review and meta-analysis of preliminary randomized controlled trials. Phys Ther.

[15] Okubo Y, Schoene D, Lord SR (2017). Step training improves reaction time, gait and balance and reduces falls in older people: a systematic review and meta-analysis. Br J Sports Med..

[16] Batcir B, Shani G, Shapiro A, Alexander N, Melzer I (2020). The kinematics and strategies of recovery steps during lateral losses of balance at different perturbation magnitudes in older adults with varying history of falls. BMC Geriatr.

[17] Smart NA, Dieberg G, Giallauria F (2013). Functional electrical stimulation for chronic heart failure: a meta-analysis. Int J Cardiol.

[18] Anderson-Hanley C, Arciero PJ, Westen SC, Nimon J, Zimmerman E (2012). Neuropsychological benefits of stationary bike exercise and a cybercycle exergame for older adults with diabetes: an exploratory analysis. J Diabetes Sci Technol.

[19] Haga M, Hoshina K, Koyama H (2020). Bicycle exercise training improves ambulation in patients with peripheral artery disease. J Vasc Surg.

[20] Verney J, Kadi F, Saafi MA, Piehl-Aulin K, Denis C (2006). Combined lower body endurance and upper body resistance training improves performance and health parameters in healthy active elderly. Eur J Appl Physiol.

[21] Nadeau A, Lungu O, Duchesne C, Robillard MÈ, Bore A, Bobeuf F, Plamondon R, Lafontaine AL, Gheysen F, Bherer L, Doyon J (2017). A 12-week cycling training regimen improves gait and executive functions concomitantly in people with Parkinson's disease. Front Hum Neurosci.

[22] Yang HC, Lee CL, Lin R (2014). Effect of biofeedback cycling training on functional recovery and walking ability of lower extremity in patients with stroke. Kaohsiung J Med Sci.

[23] Hochsprung A, Granja Domínguez A, Magni E, Escudero Uribe S, Moreno GA (2020). Effect of visual biofeedback cycling training on gait in patients with multiple sclerosis. Efectos del entrenamiento en bicicleta con retroalimentación visual sobre la marcha en pacientes con esclerosis múltiple. Neurologia.

[24] Brooke JD, Cheng J, Collins DF, McIlroy WE, Misiaszek JE, Staines WR (1997). Sensori-sensory afferent conditioning with leg movement: gain control in spinal reflex and ascending paths. Prog Neurobiol.

[25] Brown DA, Kukulka CG. Human flexor reflex modulation during cycling. J Neurophysiol. 69:1212–24, 1993. 10.1152/jn.1993.69.4.1212 [PubMed] [CrossRef] [Google Scholar]. 10.1152/jn.1993.69.4.1212 8492160

[26] Yang JF, Stein RB (1990). Phase-dependent reflex reversal in human leg muscles during walking. J Neurophysiol.

[27] Klarner T, Zehr EP (2018). Sherlock Holmes and the curious case of the human locomotor central pattern generator. J Neurophysiol.

[28] Ting LH, Kautz SA, Brown DA, Zajac FE (1999). Phase reversal of biomechanical functions and muscle activity in backward pedaling. J Neurophysiol.

[29] Batcir S, Melzer I (2018). Daily bicycling in older adults may be effective to reduce fall risks-a case-control study. J Aging Phys Act.

[30] Harvey S, Rissel C, Pijnappels M (2018). Associations between bicycling and reduced fall-related physical performance in older adults. J Aging Phys Act.

[31] Rissel C, Passmore E, Mason C, Merom D (2013). Two pilot studies of the effect of bicycling on balance and leg strength among older adults. J Environ Pub Health.

[32] Schmidt RA (1988). Motor control and learning: a behavioral emphasis.

[33] Schmidt RA, Wrisberg CA (2008). Motor learning and performance: a situation-based learning approach.

[34] Schmidt RA, Lee TD (2005). Motor control and learning: a behavioral emphasis.

[35] Shani G, Shapiro A, Oded G, Dima K, Melzer I (2017). Validity of the microsoft kinect™ system in assessment of compensatory stepping behavior during standing and treadmill walking. Eur Rev Aging Phys Act.

[36] Stahl SE, An HS, Dinkel DM, Noble JM, Lee JM (2016). How accurate are the wrist-based heart rate monitors during walking and running activities? Are they accurate enough?. BMJ Open Sport Exerc Med.

[37] Berg KO, Wood-Dauphinee SL, Williams JI, Gayton D (1989). Measuring balance in elderly: preliminary development of an instrument. Physiother Can.

[38] Swisher A, Goldfarb A (1998). Use of the six-minute walk/run test to predict peak oxygen consumption in older adults. Cardiopul Phys Ther.

[39] Cordo PJ, Nashner LM (1982). Properties of postural adjustments associated with rapid arm movements. J Neurophysiol.

[40] Takazono PS, Ribeiro de Souza C, Ávila de Oliveira J, Coelho DB, Teixeira LA. High contextual interference in perturbation-based balance training leads to persistent and generalizable stability gains of compensatory limb movements. Exp Brain Res. 2020;238(5):1249–63. 10.1007/s00221-020-05806-x. 10.1007/s00221-020-05806-x 32303810

[41] Gentile AM, Carr J, Shepherd R (2000). Skill acquisition: action, movement, and neuromotor processes in Movement Science: Foundations for Physical Therapy Rehabilitation.

[42] Melzer I, Benjuya N, Kaplanski J (2004). Postural stability in the elderly: a comparison between fallers and non-fallers. Age Ageing.

[43] Melzer I, Kurz I, Oddsson L (2010). A retrospective analysis of balance control parameters in elderly fallers and non-fallers. Clin Biomech. (Bristol, Avon).

[44] Berg KO, Wood-Dauphinee SL, Williams JI, Maki B (1992). Measuring balance in the elderly: validation of an instrument. Can J Public Health.

[45] Melzer I, Kurz I, Shahar D (2007). Application of the voluntary step execution test to identify elderly fallers. Age Ageing.

