# Supplementary material for: Development and piloting of a perturbation stationary bicycle robotic system that provides unexpected lateral perturbations during bicycling (the PerStBiRo system)
Source: BMC Geriatr. 2021 Jan 21;21:71. doi: 10.1186/s12877-021-02015-1 (PMC7818783; doi:10.1186/s12877-021-02015-1)
Supplement: Supplementary file 1 — Additional file 1: Fig. S1. Gear mechanism [file 12877_2021_2015_MOESM1_ESM.docx]

**Supplementary materials - Figure 1:** Gear mechanism
