# Supplementary material for: Development and piloting of a perturbation stationary bicycle robotic system that provides unexpected lateral perturbations during bicycling (the PerStBiRo system)
Source: BMC Geriatr. 2021 Jan 21;21:71. doi: 10.1186/s12877-021-02015-1 (PMC7818783; doi:10.1186/s12877-021-02015-1)
Supplement: Supplementary file 2 — Additional file 2: Table S1. details of each hardware component not manufactured in-house [file 12877_2021_2015_MOESM2_ESM.docx]

**Supplementary materials - Table 1:** details of each hardware component not manufactured in-house

| **Component** | **Manufacturer** | **Model** |
| --- | --- | --- |
| **Ball bearings** | NSK | Pillow Block UCP210D1 |
| **Motion controller** | Emerson | Epsilon EP202-P00-EN00 |
| **Stationary training bicycle** | BioCor | UM-3296 |
| **Servo Motor** | Emerson Control Techniques | Unimotorfm model 095E2C300VBCAA100190 |
| **Kinect camera** | Microsoft Kinect™ system | V2 |
