# Supplementary material for: Development and piloting of a perturbation stationary bicycle robotic system that provides unexpected lateral perturbations during bicycling (the PerStBiRo system)
Source: BMC Geriatr. 2021 Jan 21;21:71. doi: 10.1186/s12877-021-02015-1 (PMC7818783; doi:10.1186/s12877-021-02015-1)
Supplement: Supplementary file 3 — Additional file 3: Fig. S2. The Motion control system interactions [file 12877_2021_2015_MOESM3_ESM.docx]

**Supplementary materials - Figure 2:** The Motion control system interactions


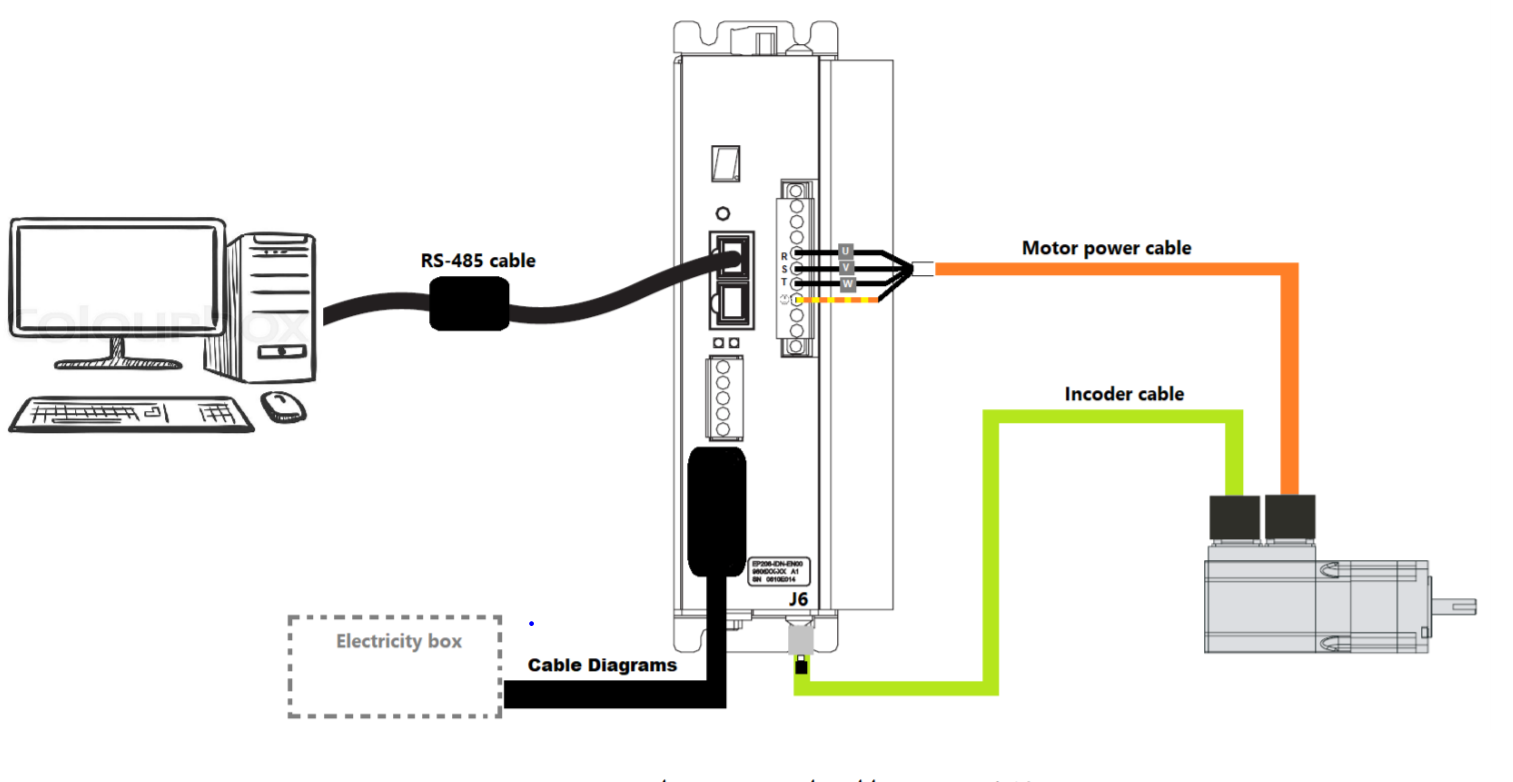


**RS-485 cable**

**C#WTF application**

**Visual Studio**

**Modbus**

**Protocol**

**Encoder cable**

**Motor power cable**

**Emerson Control Techniques Unimotor fm model 095E2C300VBCAA100190 Servo motor**

**Emerson Epsilon EP202-P00-EN00 Motion Control System**

**Electricity box**
