# Supplementary material for: Development and piloting of a perturbation stationary bicycle robotic system that provides unexpected lateral perturbations during bicycling (the PerStBiRo system)
Source: BMC Geriatr. 2021 Jan 21;21:71. doi: 10.1186/s12877-021-02015-1 (PMC7818783; doi:10.1186/s12877-021-02015-1)
Supplement: Supplementary file 4 — Additional file 4: Fig. S3. System communication flow chart. [file 12877_2021_2015_MOESM4_ESM.docx]

**Figure 3:** System communication flow chart.


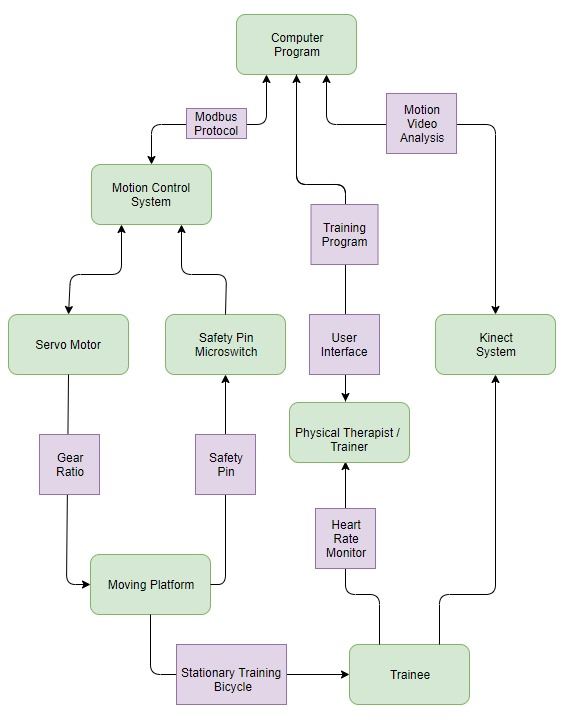


The arrows represent the propagation and directions of information and data between each part and what it is connected to. The green boxes represent the main system parts that also receive or transmit communications, while the purple boxes represent Intermediate components that help with connection or communication
